# Evaluating the Analgesic, Hemostatic, and Antimicrobial Potential of a Novel Herbal Formulation for Dental Use

**DOI:** 10.7759/cureus.84884

**Published:** 2025-05-27

**Authors:** Mariam Tariq, Amber Kiyani, Aatika Ahmed, Zainab Sohail Raja, Uzma Hassan

**Affiliations:** 1 Dental Materials, Riphah International University, Islamabad, PAK; 2 Oral Pathology, Riphah International University, Islamabad, PAK; 3 Microbiology and Pathology, Riphah International University, Islamabad, PAK; 4 Oral and Maxillofacial Surgery, Riphah International University, Islamabad, PAK; 5 Oral and Maxillofacial Surgery, Islamic International Dental College and Hospital, Islamabad, PAK

**Keywords:** caries, gingivitis, herbal medicine, oral health, periodontitis, phytomedicine, pulpitis, tannic acid, thymol, thymoquinone

## Abstract

Background: Research has explored the individual effects of phytochemicals in dentistry. Our aim was to investigate the synergistic effect these compounds have on pulpal pain, gingival bleeding, and oral microbial populations.

Methods: A proprietary formulation consisting of tannic acid, caryophyllene, curcumin, berberine, myrcene, lignin, catechin, quercetin, gallic acid, thymol, gamma-terpinene, humulene, limonene, and thymoquinone was selected for testing. The antimicrobial effect of the solution was tested using the disc diffusion method on plaque samples cultured on tryptic soy and blood agar. The hemostatic effect of the solution was evaluated by applying it to bleeding gingival tissues during the scaling procedure and recording the time until the cessation of free-flowing blood. The analgesic effect was measured through the Visual Analogue Scale (VAS) before and after topical application of the solution to the affected tooth. Time until self-reported reduction in pain was also noted.

Results: The mean zone of inhibition of the solution on tryptic soy agar and blood agar was 20 mm and 13 mm, compared to 17 mm and 15 mm of azithromycin. Colonies of *Candida*, *Streptococci*, and *Staphylococci* were identified and confirmed from the culture, showing susceptibility to the solution. The mean time until complete hemostasis was 5.8 ± 0.74 seconds. The mean pain score before application of the solution was 68 ± 11.4, and after application was 22±12.6, with a *P-*value of <0.01. The mean time for self-reported pain reduction by patients was 38 seconds.

Conclusion: The promising results from this pilot study suggest significant therapeutic efficacy, and future in-depth investigations are recommended to fully explore the potential of this novel herbal dental formulation.

## Introduction

Dental caries and periodontitis represent major global health challenges, affecting both children and adults alike, and can lead to tooth pain, bleeding gums, tooth decay, and eventual tooth loss. Despite major advances in healthcare, oral diseases affect 3.5 billion people globally, with three out of every four affected individuals belonging to middle- to low-income countries [1]. Within Pakistan, a country populated by 220 million people, 60% suffer from dental caries, with a particularly high prevalence in rural communities where access to affordable dental care remains limited [2].

Oral diseases are a well-known health burden, resulting in pain, impaired function, and reduced quality of life, with treatment costs serving as a significant burden for both individuals and the healthcare system [1]. Antibiotics and analgesics are frequently used as initial treatments; however, overuse and misuse by both patients and practitioners have led to adverse effects and a growing concern of antimicrobial resistance [3,4]. This underscores the need for safe and effective alternatives to conventional synthetic medications.

For centuries, people have relied on plant-based medicines, whose therapeutic potential is attributed to the presence of secondary metabolites that possess a wide range of pharmacological properties. These medicinal plants have been used alone or as adjuncts to synthetic medications to treat a wide range of illnesses [5]. Moreover, in recent times, the popularity of phytomedicine has increased significantly due to its affordability, accessibility, ease of use, cost-effectiveness, and reduced side effects compared to synthetic drugs [6].

Traditionally, various plant-based medicines have been used to treat oral diseases, including those derived from miswak, olive leaf extract, and pomegranate [7]. The therapeutic efficacy of many such remedies has been validated through in vitro and in vivo studies [8]. Extracts of many different plants show an inhibitory effect against several oral pathogens responsible for tooth demineralization and periodontal disease, such as *Streptococcus mutans*,* Porphyromonas gingivalis*,and* Candida albicans* [8]. Additionally, herbal remedies have shown potential in reducing the symptoms of periodontal disease, alleviating halitosis and mouth discomfort, and encouraging bone regrowth and wound healing [7].

Research has shown that combining multiple plants and phytochemicals, either with each other or with synthetic drugs, can enhance the overall therapeutic effect, a principle known as synergism [9]. This synergistic approach, combined with the urgent need for effective oral care solutions in Pakistan, prompted our search for a plant-derived oral formulation. We identified Dentizin™, manufactured by Remedius Pharma Pvt. Ltd. (Islamabad, Pakistan), as a phytochemical formulation for dental care, containing a mixture of bioactive compounds, including tannic acid, caryophyllene, curcumin, berberine, myrcene, lignin, catechin, quercetin, gallic acid, thymol, gamma-terpinene, humulene, limonene, and thymoquinone. To the best of our knowledge, no other formulation using this specific combination of phytochemicals has been reported.

The goal of this pilot study was to evaluate the therapeutic potential of the abovementioned phytochemical formulation in the context of oral health and to gather preliminary data regarding its efficacy. We investigated the antimicrobial properties against oral bacteria, hemostatic effects on gingival tissue, and analgesic effects on pulpal pain.

## Materials and methods

The Ethical Review Board at Riphah International University provided ethical approval (IIDC/IRC/2023/004/01/012) for this open pilot study, which was conducted over a six-month duration at Riphah International Hospital. Participants presenting to the Dental Outpatient Department were selected using convenience sampling. All participants were above the age of 12. Prior to obtaining consent, the participants were briefed on the purpose of the study and the herbal nature of the solution. Additionally, they were informed that all measures to maintain confidentiality and anonymity during the study process and data publication would be implemented.

In vitro effects

This portion of the study aimed to evaluate the antimicrobial activity of the solution against a comprehensive range of oral bacteria present in plaque and saliva samples. Ointments, gels, and rinses for the oral cavity must be active against a diverse population of microbes that influence each other's growth. Therefore, we used an initial screening approach using the disc diffusion method to determine if the solution was generally effective against microbes obtained from oral plaque and saliva samples, followed by the identification of susceptible microorganisms [10].

Preparation of Test Medium

A 10 mL bottle of Dentizin™ was poured into a sterile glass petri dish and allowed to dry under a laminar flow hood overnight. The dried extract was scraped off and re-dissolved in 1.5 mL of 99% ethanol (Sigma-Aldrich, USA). Filter paper discs (6 mm) were prepared using Whatman No. 1 filter paper and dipped into the mixture for 10 seconds. The discs were then air-dried on a sterile surface to allow the ethanol to evaporate. Care was taken to ensure that the solution was not exposed to natural light and that sterility was maintained throughout the process.

Isolation of Bacteria

Patients presenting with clinical signs and symptoms of periodontal disease, including gingival inflammation (redness and swelling), a bleeding on probing (BOP) score of ≥40%, and visible plaque and calculus deposits, were chosen for microbial samples [11]. A probe was then inserted into the gingival crevice on the lingual surface of the mandibular incisors, selected for its ease of access, to separate the subgingival plaque. Then, plaque and saliva samples were obtained using a sterile cotton swab and immediately spread onto three plates of tryptic soy agar (TSA) and blood agar.

The disc diffusion method was used to determine the antimicrobial efficacy of the solution [12]. Three discs were placed onto each agar plate. As positive and negative controls, a disc infused with 15 μg of azithromycin (Liofilchem, Italy) and distilled water was used and compared to a prepared disc infused with Dentizin™. The tryptic soy plates were inverted and incubated at 37 °C in an aerobic incubator. The blood agar plates were placed in an anaerobic chamber [13]. After 18 hours, the widest zones of inhibition around the discs were measured (including the diameter of the disc) using a Vernier caliper and rounded to the nearest millimeter. The entire test was performed in triplicate using total samples from nine different patients.

Identification of Microorganisms

Based on their appearance, colonies consistent with Streptococci, Staphylococci, and Candida were picked from the agar plates and subcultured on new plates of tryptic soy and blood agar. Microorganisms were confirmed using Gram staining and periodic acid-Schiff (PAS) staining [14].

In vivo effects

Hemostatic Effect

Forty patients presenting with clinical signs of periodontal disease, including gingival inflammation (redness and swelling), a BOP score of ≥40%, and visible plaque and calculus deposits, were included in this portion of the study. The BOP level ensured a baseline level of gingival inflammation in all patients. Additionally, those with latent or active HIV/Hep C infection, a history of cardiovascular disease or bleeding disorders, those taking anticoagulants, or those suffering from any systemic disease that could affect oral health were excluded.

Selected patients then underwent routine scaling and root planing. The test solution was applied during the scaling procedure only on sites with active, free-flowing bleeding during the procedure. A small rolled piece of cotton (approximately 1 × 1 cm) soaked in four drops of the test solution was held gently against the gingival margin using forceps and intermittently removed to visually assess bleeding cessation. Four drops were used to ensure a consistent amount for all patients and to adequately saturate the small cotton piece. The duration from the time the solution was applied to the cessation of free-flowing blood was recorded using a digital chronometer [15]. Therefore, the hemostatic effect focused on time until the cessation of bleeding and not on the initial intensity. The patients were recalled on day one of follow-up to assess for any side effects or allergic reactions. Results were presented as mean value with standard deviation.

Analgesic Effect

Fifty consenting patients from the Dental Outpatient Department with pulpal pain lasting more than a week and a pain score of more than 50 mm on the Visual Analog Scale (VAS) were selected [16]. Patients under the age of 12, those with systemic disease, or those who had taken analgesic/anti-inflammatory medication in the last 12 hours were excluded from the study. The 12-hour cutoff was chosen to avoid the influence of recent medication on pain perception, as many drugs for dental pain, such as ibuprofen, provide relief for up to six hours and naproxen for 8-12 hours [17]. This ensured that the baseline pain levels measured were not affected by any residual analgesic effects.

A detailed history of pain was recorded. Clinical examination was conducted using a mirror, percussion test, and a triple syringe to identify the affected tooth. Teeth that showed clinical or radiographic evidence of abscess or periodontal pathology were excluded. After identification of the tooth, the severity of pain was measured using the VAS. A piece of cotton, small enough to fit into the cavity and soaked in four drops of the solution, was then placed directly into the tooth for one minute. The amount of solution used was sufficient to soak the cotton piece and was chosen to maintain consistency across all participants, following the approach of studies that use fixed doses to ensure reliable outcomes [18]. Pain intensity was reassessed using the VAS, and patients were asked to signal when they experienced substantial pain relief by raising their hands. This time was recorded, and a cotton roll in the vestibule prevented salivary contamination. All patients received routine standard dental care after testing.

Statistical analysis

Statistical analysis was performed using IBM SPSS Statistics for Windows, Version 29 (Released 2023; IBM Corp., Armonk, New York). The mean values and standard deviation were calculated for antimicrobial zones of inhibition, hemostatic time, and pain reduction scores. In addition, a paired t-test was used to compare pre- and post-treatment values for the analgesic effect to determine the significance of the results. The *P*-value was set at 0.05.

## Results

Antibacterial effect

**Figure 1:**
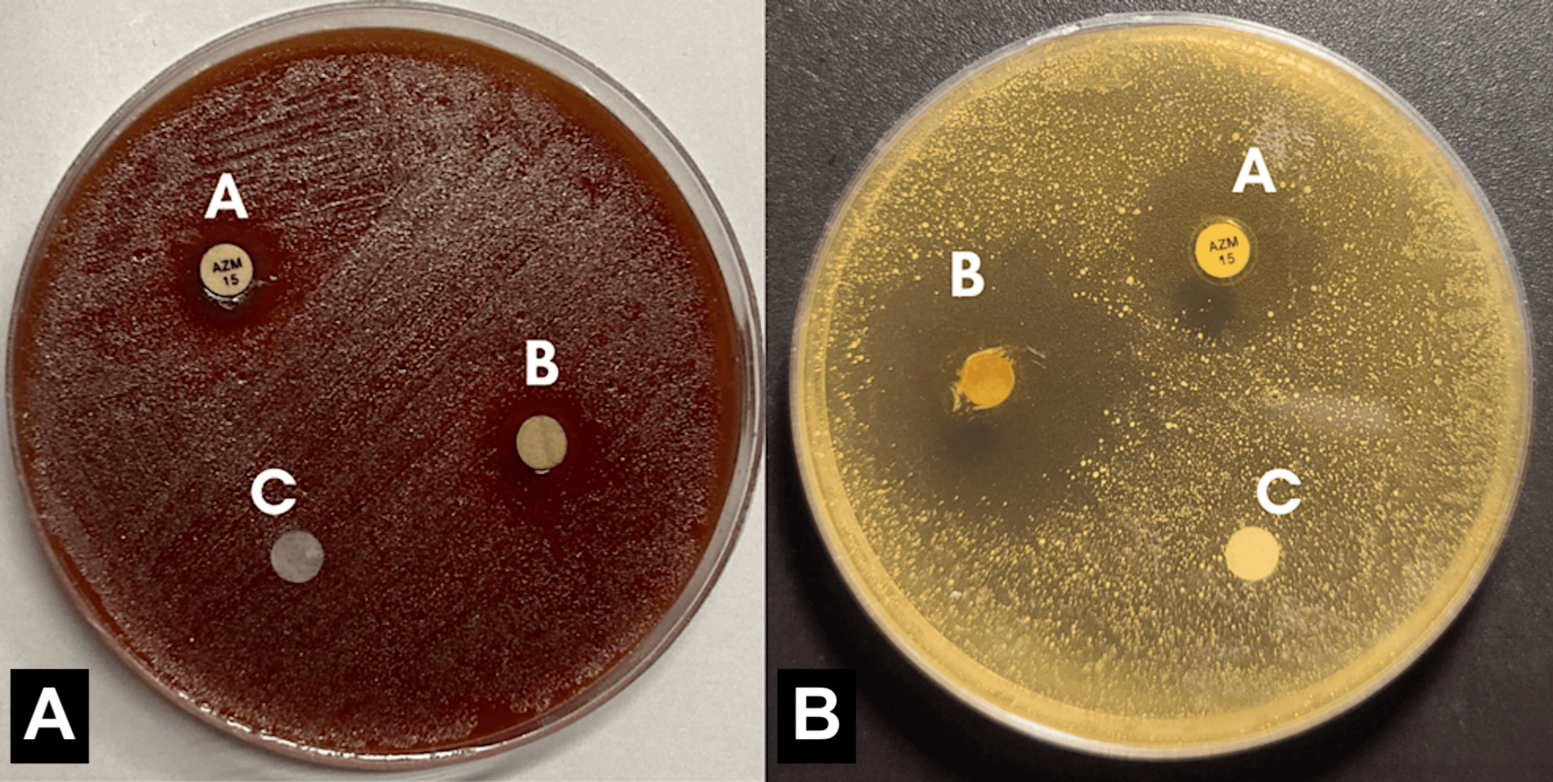
Disc diffusion testing on (A) blood agar and (B) tryptic soy agar Discs labeled as A: positive control (azithromycin), B: Dentizin™, C: negative control (distilled water).

The solution showed a significant antimicrobial effect against a general culture of microorganisms obtained from plaque and saliva (Table 1), which was comparable to azithromycin on TSA (aerobic) and blood agar (anaerobic), as shown in Figure 1. Colonies of *Streptococcus *spp.,* Staphylococcus *spp.,and* Candida *spp. that showed susceptibility to the solution were subcultured onto new plates and identified through microscopic staining.

**Table 1:** Disc diffusion assay of azithromycin and Dentizin™ against plaque and saliva cultures TSA: tryptic soy agar; SD: standard deviation

Agar	Mean zone of inhibition (mm) with SD	
	Azithromycin	Dentizin™
TSA agar	17 ± 1.2	20 ± 3.1
Blood agar	15 ± 2.1	13 ± 2.3

Hemostatic effect

**Figure 2:**
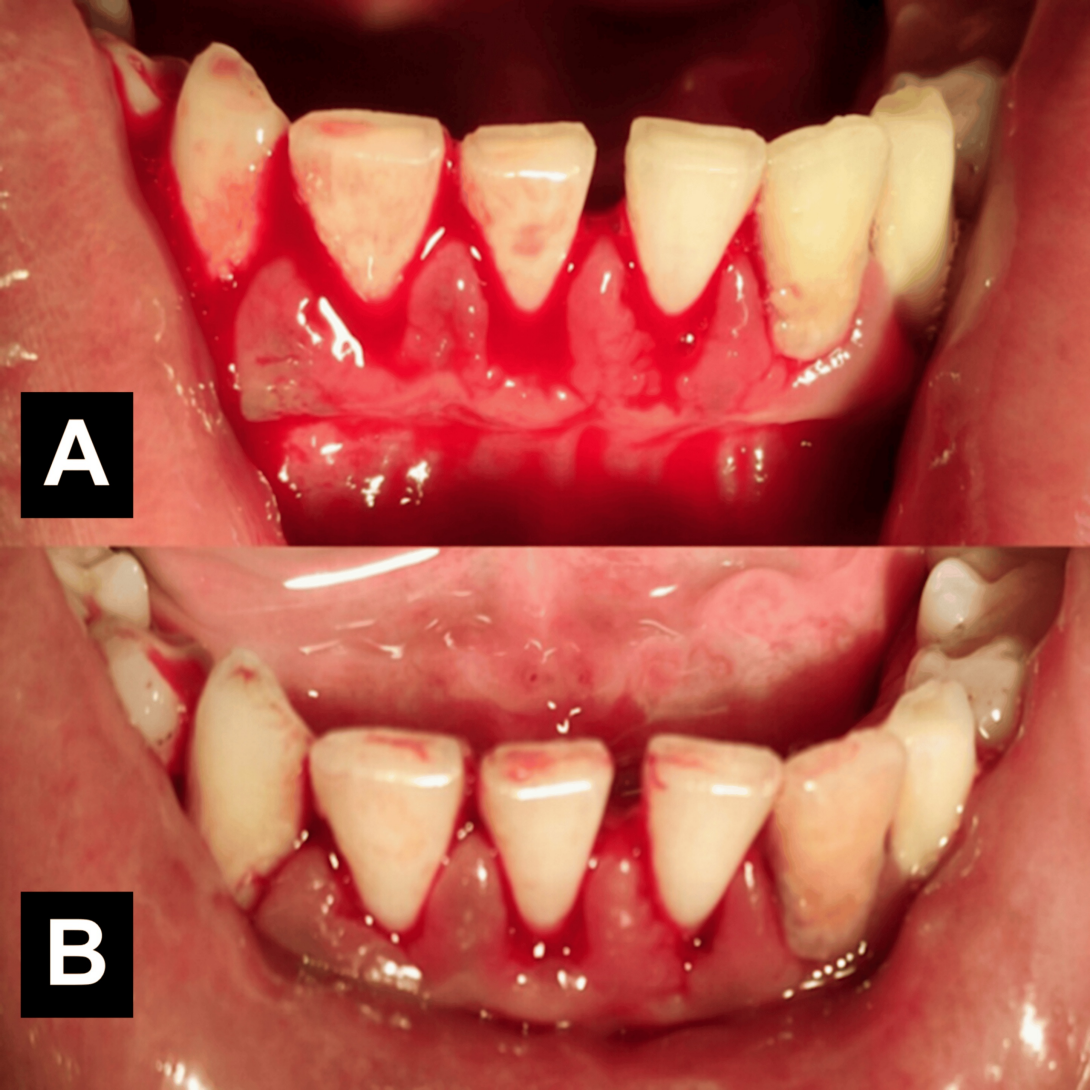
Hemostatic effect of Dentizin™ on gingival bleeding (A) Bleeding gingival tissues during the scaling procedure of a periodontally diseased patient; (B) cessation of bleeding after five seconds of application.

The average time for cessation of gingival bleeding using Dentizin™ was 5.8 seconds (Table 2). Figure 2 shows the gingival tissues before and after the application of the solution. A recurrence of bleeding did not occur until tissues were aggravated using a sharp instrument, and there were no reported adverse effects or allergies on day one follow-up.

**Table 2:** Hemostatic activity of Dentizin™ on gingival bleeding during the scaling procedure

Variable	Result
Mean time for hemostasis	5.8 seconds
Minimum value	5 seconds
Maximum value	7 seconds
Standard deviation	0.74

Analgesic effect

The mean pain score using the VAS was reduced from 68 to 22 after 60 seconds of application of the solution (Table 3), with a *P*-value of <0.01, indicating a 67% reduction in pain. On average, patients signaled substantial relief in tooth pain after 38 seconds.

**Table 3:** Analgesic effect by direct application of Dentizin™ into the tooth cavity for one minute, measured using the VAS VAS: Visual Analog Scale; SD: standard deviation

Activity	Mean pain with VAS (millimeters)	SD Pain with VAS (millimeters)
Before application of Dentizin™	68	11.4
After application for 60 seconds	22	12.6

## Discussion

The present study evaluated the therapeutic potential of a phytochemical formulation in dentistry by assessing its antimicrobial, hemostatic, and analgesic effects. The antimicrobial activity was tested using the disc diffusion method on microorganisms from a dental plaque sample, showing inhibition zones of 20 mm and 13 mm under aerobic and anaerobic conditions, respectively. From the growth on these discs, *Streptococcus, Staphylococcus, and Candida *species were identified. Additionally, the formulation achieved hemostasis in an average of five seconds and provided analgesic effects within 38 seconds.

The therapeutic effects of this formulation can be attributed to the synergistic actions of its constituents. Tannic acid, a plant-based polyphenol, has shown hemostatic properties both in vitro and in vivo and has been recommended for use in wound dressings [19]. It can also reduce the viability of non-adherent bacteria in both saliva and biofilms, along with the precipitation of salivary proteins required for the initial bacterial adherence to teeth [20]. Other formulation components, including gallic acid and quercetin, have demonstrated antimicrobial effects against various oral pathogens, including *Streptococcus mutans*, which is responsible for tooth demineralization [21,22]. Additionally, curcumin has been shown to inhibit *Porphyromonas gingivalis*, a major contributor to periodontal disease [23].

Thymol, a constituent of thyme, is an active ingredient in several commercially available mouthwashes, most notably Listerine, and has been shown to reduce plaque levels and gingivitis [24]. Additionally, several constituents of the formulation contribute to analgesic effects, such as berberine and quercetin, which modulate pain pathways by inhibiting inflammatory mediators [25,26].

To provide clinical context, the solution's hemostatic effect compares favorably with ferric chloride, which is used for gingival tissue displacement but requires an application time of three to five minutes, with reports of corrosive injury to soft tissue, though direct comparative studies are needed to confirm equivalence in clinical settings [27]. Additionally, the formulation demonstrated a 20 mm inhibition zone against mixed oral microbes on blood agar, showing comparable antimicrobial activity to 0.2% chlorhexidine, which matched its efficacy against Streptococcus mutans (20.85 ± 1.18 mm) and exceeded its activity against *Candida albicans* (12.4 ± 0.6 mm) [28].

The development of a safe and effective plant-based formulation for dental care is crucial worldwide, particularly in countries like Pakistan, where more than half of the population suffers from dental caries. In addition, a lack of dentists, coupled with the unaffordability of private treatment and low oral health literacy, leads to reduced care, with individuals only seeking treatment at advanced stages of the disease due to severe symptoms of pain [29]. Therefore, there is a widespread trend of self-prescription of antibiotics and painkillers [30]. This issue is worsened by lax pharmaceutical regulations and easy access to prescription medications [31].

To minimize potential bias, data collection was conducted by three independent investigators. Objective measurement tools were employed throughout the study, including a digitally calibrated chronometer for precise timing of hemostasis and a VAS for consistent pain assessment. Additionally, we acknowledge the lack of negative controls in both the hemostatic and analgesic components of this study. The test solution had a dark brown color along with a strong aroma and taste, making effective blinding and the use of a placebo difficult. Moreover, this was a pilot study aimed at observing preliminary effects in a clinical setting. Similar pilot studies involving herbal preparations have been published without control groups; for example, a study assessing the effects of herbal supplements on post-COVID-19 fatigue and a prospective pilot study in which all participants with chronic hepatitis C received birch bark extract for 90 days, with outcomes measured as changes from baseline [32,33]. These studies, like ours, prioritized initial observational insights to guide future controlled trials.

## Conclusions

This pilot study investigated the therapeutic potential of a novel phytochemical formulation for oral care. It indicated that the solution effectively inhibited oral microbial populations, controlled gingival bleeding within six seconds, and reduced pulpal pain scores by 67%, with patients reporting relief in under a minute. No adverse effects were reported.

Given the high burden of dental diseases and limited access to professional dental care in many areas of Pakistan, this natural formulation may provide a complementary option for managing oral disease symptoms in Pakistan, particularly in areas with limited dental care access. While not intended to replace standard treatments, it could help reduce inappropriate self-medication with antibiotics and painkillers. As this was a preliminary pilot study, further controlled trials are needed to validate its efficacy, long-term safety, and appropriate applications as an adjunct to conventional dental care.
